# Scar Pregnancy a Diagnostic Conundrum: A Case Report

**DOI:** 10.31729/jnma.5202

**Published:** 2021-03-31

**Authors:** Manoj Pokhrel, Shreedhar Prasad Acharya, Jyotshna Sharma, Meena Thapa

**Affiliations:** 1 Department of Obstetrics and Gynaecology, Kathmandu Medical College and Teaching Hospital, Sinamagal, Kathmandu, Nepal; 2 Kathmandu Medical College and Teaching Hospital, Sinamagal, Kathmandu, Nepal

**Keywords:** *cesarean section*, *laparotomy*, *scar prganancy*

## Abstract

Cesarean scar pregnancy is a rare form of ectopic pregnancy which may lead to uterine rupture and catastrophic hemorrhage. We report a case of cesarean scar pregnancy in a 35-year-old female with the past history of cesarean section presented with complaints of amenorrhoea for 6 weeks and non-specific periumbilical pain. Two Transvaginal sonography was done 48 hours apart which suggested a cesarean scar pregnancy in one and cervical pregnancy on the other. Magnetic Resonace Imaging showed a well-defined cystic lesion of (21x19)mm^2^ embedded within the previous cesarean scar which confirmed the diagnosis of cesarean scar pregnancy. Laparotomy unveiled uterus around 6 weeks size and a (3x3)cm^2^ bulge was noted at the site of previous scar in lower uterine segment, where a small incision was given and the gestational sac was removed following which the uterine incision was closed with 2-0 polyglactin suture. High index of suspicion and prompt diagnosis is of paramount for reducing morbidity and mortality.

## INTRODUCTION

Cesarean scar pregnancy (CSP) is a rare form of ectopic pregnancy in which the gestational sac is completely or partially implanted in the uterine scar of the previous cesarean section (CS).^1^ The reported incidence is varied ranging from 1 in 1800-2500 pregnancies.^2,3^ CSP is commonly mistaken for cervical ectopic pregnancy and abortion.^4^ Delay in diagnosis may lead to uterine rupture and catastrophic hemorrhage leading to morbidity and mortality.^5^

We present a case of a 35-year-old woman with previous CS who was asymptomatic and was diagnosed as CSP not without a diagnosis dilemma. She was treated surgically and had an uneventful recovery.

## CASE REPORT

A 35-year-old G_2_P_1_ with history of previous cesarean section attended the Out Patient Department (OPD) of Kathmandu Medical College with history of amenorrhea for one and half months, and non-specific periumbilical pain with no other alarming symptoms. On examination her general condition was fair, her vitals were within normal limits. Per abdominal examination and per speculum failed to reveal any abnormality.

**Figure 1A, 1B.**
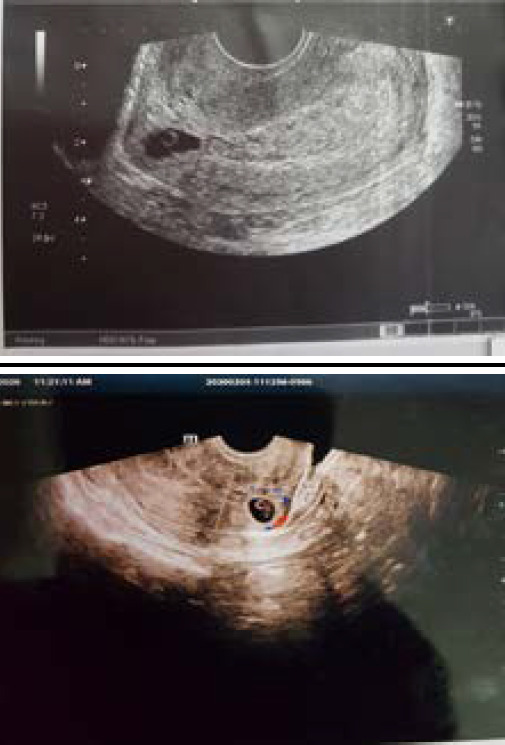
Transvaginal sonography 1st (1A) suggestive of scar pregnancy, Second Transvaginal sonography 2 days after the first Transvaginal sonography suggestive of cervical pregnancy (1B).

She however produced two transvaginal sonography (TVS) reports, one report revealed a single gestational sac of 6 weeks 1 day in the anterior myometrium with subtle echogenicity in the adjacent wall suggestive of scar pregnancy (Figure 1A). The other TVS done a couple of days apart showed an intrauterine gestational sac corresponding to 6 weeks 5 days in the uterine endocervix which suggested a cervical pregnancy (Figure 1B).

**Figure 2.**
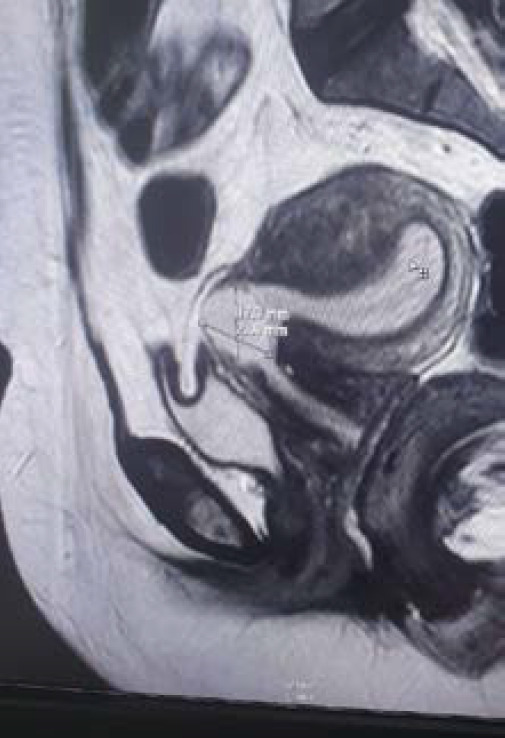
MRI showed a well-defined cystic lesion of 21 X 19mm^2^ in the anterior aspect of the lower uterine segment embedded within the previous cesarean scar.

She was admitted and planned for an Magnetic Resonance Imaging (MRI) of pelvis due to the diagnostic dilemma. MRI conclusively showed a well-defined cystic lesion of (21x19)mm^2^ in the anterior aspect of the lower uterine segment embedded within the previous cesarean scar evocative of cesarean scar pregnancy (Figure 2).

**Figure 3A, 3B:**
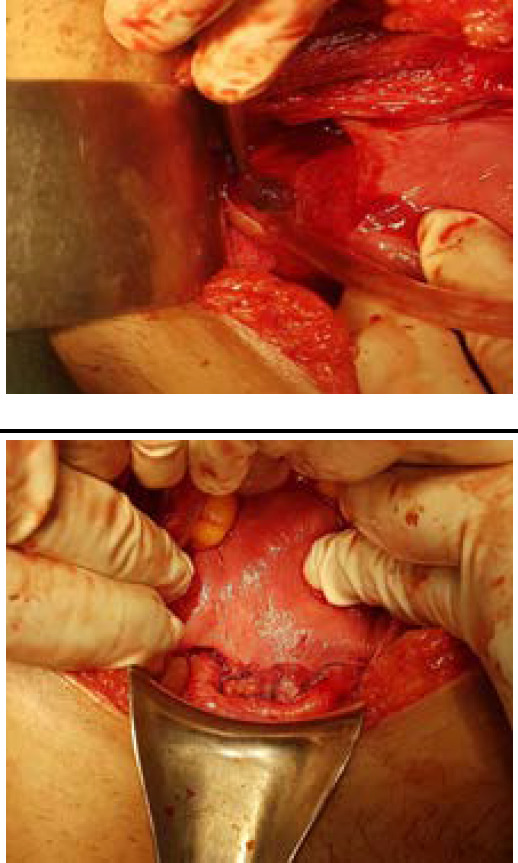
A small bulge of 3 X 3cm^2^ was noted at the lower uterine segment (3A). Closed uterine incision with 2-0 polyglactin suture after the removal of gestational sac (3B).

With the provisional diagnosis of cesarean scar pregnancy, laparotomy was performed. Laparotomy unveiled uterus around 6 weeks size, bilateral ovaries and fallopian tubes were normal. However a (3x3)cm^2^ bulge was noted at the lower uterine segment at the site of previous cesarean scar (Figure 3A). Previous scar was intact and a small incision was given over the bulge and the gestational sac was removed following which the uterine incision was closed with 2-0 polyglactin suture (Figure 3B).

She had an uneventful postoperative period and was discharged on the 4^th^ postoperative day on oral antibiotics.

## DISCUSSION

CSP is the rarest kind of ectopic pregnancy^1^ but the incidence is rising attributed to the increasing trend of cesarean delivery, increasing awareness and better diagnostic ultrasonography. The incidence of CSP is around 6% of ectopic pregnancies among women with previous cesarean section, however this does not seem to correlate with number of cesarean sections.^4,5^ The mechanism and etiopathogenesis remains obscure, however the migrating blastocyst implants into the scar as a result of invasion into the wedge defect or microscopic fistula from the trauma inflicted by the earlier surgery.^6^

The patients can present to the clinicians with a variety of symptoms, ranging from minimal vaginal bleeding and abdominal discomfort to severe abdominal pain and hypovolemic shock.^7,8^ Our case was presented to us early in 6th week of gestation, this might be the reason that the case was asymptomatic and was diagnosed incidentally. The diagnosis in our case was not straightforward, she had to undergo various diagnostic modalities consisting of abdominal USG, TVS and MRI until we reached a probable diagnosis.

A high index of suspicion and detailed ultrasonographic assessment is necessary for diagnosis. Ultrasonography is the main modality for diagnosis. Combined trans-abdominal and trans-vaginal ultrasound yields accurate results.^9^ MRI is used as an adjunct for the diagnosis in cases with fibroid uterus and in advanced gestational age or when ultrasound is inconclusive.

The ultrasound criteria for diagnosis of CSP are empty uterine cavity and cervical canal, placenta and or gestational sac embedded in the scar of the previous cesarean, a triangular or oval/round gestational sac that fills the niche of the scar, a thin or absent myometrial layer between the gestational sac and the bladder, evidence of functional trophoblastic/ placental circulation on color flow Doppler, characterized by high velocity and low impedance flow and negative ‘sliding organ’ sign.^10^

There is no universal treatment guideline formulated till date. Studies have shown various treatment modalities ranging from expectant management, medical management to surgical procedures.

However, management depends upon various variables such as patient factor which includes symptoms, fertility wishes and associated risk factors. Treatment depends upon the gestational age, type and size of CSP and level of human chorionic gonadotrophin (hCG), finally management also depends upon the surgical expertise and facility of the center.

Methotrexate is widely used as medical therapy in CSP, it can be administered locally as well as systemically, in single dose or multi dose regime. However its success depends on the age of gestation and the value of hCG. The success rate was higher when the hCG value was less than 5000U/L and gestational age was less than 8 weeks. Case reviews showed that 41% of cases resolved with the use of systemic methotrexate alone.^11^

This case was diagnosed incidentally and was hemodynamically stable thus we performed a hysterotomy and removed the products of conceptus and repaired the uterus hence fertility was preserved and intraoperative or postoperative morbidity was significantly reduced.

Ultrasound guided dilatation and curettage is a commonly used surgical treatment option in case of endogenous CSP with myometrial thickness of over 2mm, however there is a risk of hemorrhage and incomplete procedure.

The advantage of surgical resection is complete removal of the product of conceptus with appropriate repair of uterine defect. Surgical resection can be achieved via laparoscopy or laparotomy depending upon the expertise in case of exogenous CSP. High index of suspicion and prompt diagnosis and individualized treatment is of paramount for reducing the morbidity and mortality.
